# When MINMOD Artifactually Interprets Strong Insulin Secretion as Weak Insulin Action

**DOI:** 10.3389/fphys.2021.601894

**Published:** 2021-04-22

**Authors:** Joon Ha, Ranganath Muniyappa, Arthur S. Sherman, Michael J. Quon

**Affiliations:** ^1^Laboratory of Biological Modeling, National Institute of Diabetes and Digestive and Kidney Diseases, National Institutes of Health, Bethesda, MD, United States; ^2^Diabetes, Endocrinology and Obesity Branch, National Institute of Diabetes and Digestive and Kidney Diseases, National Institutes of Health, Bethesda, MD, United States

**Keywords:** minimal model, hyperinsulinemic euglycemic clamp, insulin resistance, racial disparities, insulin secretion

## Abstract

We address a problem with the Bergman-Cobelli Minimal Model, which has been used for 40 years to estimate *S*_*I*_ during an intravenous glucose tolerance test (IVGTT). During the IVGTT blood glucose and insulin concentrations are measured in response to an acute intravenous glucose load. Insulin secretion is often assessed by the area under the insulin curve during the first few minutes (Acute Insulin Response, AIR). The issue addressed here is that **we have found in simulated IVGTTs, representing certain contexts, Minimal Model estimates of *S*_*I*_ are inversely related to AIR, resulting in artifactually lower *S*_*I*_.** This may apply to Minimal Model studies reporting lower *S*_*I*_ in Blacks than in Whites, a putative explanation for increased risk of T2D in Blacks. The hyperinsulinemic euglycemic clamp (HIEC), the reference method for assessing insulin sensitivity, by contrast generally does not show differences in insulin sensitivity between these groups. The reason for this difficulty is that glucose rises rapidly at the start of the IVGTT and reaches levels independent of *S*_*I*_, whereas insulin during this time is determined by AIR. The minimal model in effect interprets this combination as low insulin sensitivity even when actual insulin sensitivity is unchanged. This happens in particular when high AIR results from increased number of readily releasable insulin granules, which may occur in Blacks. We conclude that caution should be taken when comparing estimates of *S*_*I*_ between Blacks and Whites.

## Introduction

The Minimal Model (MINMOD) has been a resounding success by any measure. The original paper (Bergman et al., 1979) has been cited over 2,000 times, and the numerous variants of the model developed by the Cobelli group have been cited collectively many thousands of times. MINMOD was designed to measure insulin sensitivity (*S*_*I*_) during an intravenous glucose tolerance test (IVGTT) by fitting the glucose response following an injected bolus of glucose, with the measured insulin used as a model input.

Acute intravenous injection of glucose stimulates the release of insulin mainly from the rapidly releasable pool (RRP) within the beta cells. The area under the curve of plasma concentrations of insulin during the first 10 min, termed the acute insulin response (AIR) is often used as a measure of insulin secretion (Cobelli et al., 2007). AIR varies inversely with *S*_*I*_, reflecting the compensatory increase of insulin secretion to compensate for deteriorating insulin sensitivity (Bergman et al., 1981; Cobelli et al., 2007). Our main finding is that MINMOD may underestimate *S*_*I*_ when AIR is large and therefore be unreliable in comparing *S*_*I*_ between groups with very different characteristic levels of AIR. This is distinct from the fundamental observation that *S*_*I*_ and AIR tend to vary inversely. When the product *S*_*I*_^∗^AIR, known as the Disposition Index (DI), is nearly constant as *S*_*I*_ decreases, i.e., when insulin secretion, represented by AIR increases in proportion, normoglycemia is maintained. In contrast, DI decreases as individuals progress from normal glucose tolerance through impaired glucose tolerance to type 2 diabetes (T2D) (Bergman et al., 1981; Cobelli et al., 2007). This concept is a cornerstone of the modern understanding of T2D pathogenesis, as it makes quantitative the concept that T2D is avoided if insulin secretion (beta-cell function) increases in inverse proportion to falling insulin sensitivity but occurs if the beta cells are unable to mount such a compensatory response. Here we consider a case in which a group with higher DI paradoxically has higher risk of T2D, potentially casting doubt on the DI paradigm.

Our starting point and motivation are the published observations from many groups that Blacks have lower *S*_*I*_ than Whites when assessed by MINMOD (Haffner et al., 1996, 1997; Festa et al., 2006; Goedecke et al., 2009; Goree et al., 2010; Kodama et al., 2013). This deficit is a possible explanation for the greater risk of T2D among Blacks. However, other studies of insulin sensitivity using the reference hyperinsulinemic euglycemic clamp method (HIEC) have by and large not found differences between Blacks and Whites (Saad et al., 1991; Stefan et al., 2004; Pisprasert et al., 2013; Ebenibo et al., 2014; Bello et al., 2019) which suggests that the enhanced risk of T2D among Blacks lies elsewhere. Resolving the discordance between these two well-established techniques of assessing insulin sensitivity is important for designing clinical trials and therapies optimized for preventing and treating T2D among Blacks.

IVGTT studies also show that Blacks have higher AIR and higher DI (Kodama et al., 2013), which should be protective against T2D, but nonetheless have higher T2D risk. This is a paradox that we will not attempt to resolve in this limited study. Rather, we will examine closely the relationship between AIR and *S*_*I*_ with a goal of determining which set of observations and interpretations to credit.

The equations for MINMOD, as implemented in MINMOD Millennium (Boston et al., 2003), are:

(1) $$\frac{dG}{dt}=G_{b}S_{G}-\left(S_{G}+X\right)G$$

(2) $$\frac{dX}{dt}=-p_{2}X+p_{3}\left(I-I_{b}\right)$$

where the independent variables are glucose, *G*, and insulin action, *X*, taken to be proportional to insulin in a remote (interstitial) compartment, which is not measured but estimated along with *G* using the measured *I* values as input to the model. The other measured quantities are basal glucose, *G_b_*, and basal insulin, *I_b_*.

By fitting *G*, the model estimates parameters *p*_2_ and *p*_3_, which are combined to yield an estimate of insulin sensitivity *S_I_*, defined as *p*_3_/*p*_2_. Finally, parameter *S_G_* is estimated and interpreted as the ability of glucose to promote its own uptake independent of insulin (glucose effectiveness).

We have previously described a model for longitudinal diabetes progression (Ha et al., 2016; Ha and Sherman, 2020) that builds on the core physiology represented by Eqs. 1, 2. That model was shown to be able to represent responses at any stage of glycemic progression during IVGTTs and oral glucose tolerance tests (OGTTs). Our approach will be to use that model (referred to here as the synthetic model) to generate responses of virtual individuals with prescribed parameters for insulin sensitivity and beta-cell function and investigate how well MINMOD and HIEC recover the assumed parameters.

## Materials and Methods

The synthetic model described here was developed to describe the pathogenesis of type 2 diabetes over months and years (Ha et al., 2016) and then extended to simulate oral glucose tolerance tests (OGTTs) and IVGTTs at fixed time points during that process (Ha and Sherman, 2020). Here we employ the model to generate virtual individuals for use in testing the ability of MINMOD and HIEC to estimate parameters of insulin resistance for subjects with defined characteristics. Terms and symbols are listed in Table 2.

Following (Topp et al., 2000) we first rewrite the glucose equation for MINMOD as:

$$\frac{dG}{dt}=R_{0}-\left(S_{G}+X\right)G$$

where *R*_0_ can be viewed as the input of glucose to the plasma compartment from either exogenous sources, such as intravenous injection and absorption from the gut, or from endogenous glucose production. Whereas glucose input is constant in MINMOD, we make it time-dependent and add more physiological detail. First, we subdivide *R*_0_ into exogenous and endogenous terms:

$$R_{0}=R_{exo}+R_{endo}$$

For IVGTTs, *R*_*exo*_ is a function that rises and decays sharply:

$$R_{exo}\left(t\right)=\frac{IVGTT_{bar}}{BWV_{G}}t^{\kappa }\exp\left(-\lambda t\right)$$

Here *BW* is body weight, *V*_*G*_ is the volume of distribution for glucose, and *IVGTT*_*bar*_ sets the scale of the total glucose bolus. The parameters for *R*_*exo*_ are fixed in this study and are listed in the Supplementary Material—Equations, Supplementary Table 3.

For OGTTs, we use a piece-wise linear function, simplified from the formula in Dalla Man et al. (2002), that rises and falls more gradually than in the IVGTT due to slow absorption from the gut:

$$\begin{array}{c} R_{exo}\left(t\right)=\left(a_{i-1}+\frac{a_{i}-a_{i-1}}{t_{i}-t_{i-1}}\left(t-t_{i-1}\right)\right)/V_{G},t_{i-1}<t<t_{i},\\ \;i=1,2,3\;\mathrm{and}\;0\;\mathrm{elsewhere}. \end{array}$$

where *V_G_* is the volume of distribution for glucose. The parameters for *R*_*exo*_ are fixed in this study and are listed in the Supplementary Material—Equations, Supplementary Table 2.

The formula for *R*_*endo*_, representing mainly hepatic glucose production (HGP) is:

$$R_{endo}\left(t,I,S_{I}\right)=\frac{hepa_{max}\left(S_{I}\right)}{\alpha_{HGP}\left(S_{I}\right)+hepa_{SI}I}+HGP_{bas}$$

*R*_*endo*_ is a decreasing function of *I* that depends on *S_I_* to account for the correlation between hepatic and peripheral insulin sensitivity and on *hepa*_*SI*_ to account for a component of hepatic-specific insulin sensitivity independent of *S_I_*. The only parameter varied in this study is *S_I_*. The details of *hepa*_*max*_ and α_*HGP*_(*S*_*I*_) are in the Supplementary Material—Equations, Eqs. A5, A6 and the fixed parameters are in Supplementary Table 4.

We rewrite the glucose equation compactly, showing only the parameters of *R*_0_ that are varied in this study:

(3) $$\frac{dG}{dt}=R_{0}\left(t,I,S_{I}\right)-\left(S_{G}+X\right)G$$

We have added an equation for *X* to the model in Ha and Sherman (2020) to more accurately represent IVGTTs. It is the same as in MINMOD, but with *p*_2_ factored out to show *S*_*I*_explicitly:

(4) $$\frac{dX}{dt}=-\left[X-S_{I}\left(I-I_{b}\right)\right]/p_{2}$$

The synthetic model adds an equation for insulin, which we use to generate virtual subject with different capacities to secrete insulin and hence different AIR when assessed by IVGTT. It represents the balance between secretion rate, *ISR*, and clearance:

(5) $$\frac{dI}{dt}=\frac{\beta }{V}ISR\left(\gamma ,\sigma ,r_{2}^{0}\right)-kI$$

where *V* is the volume of distribution for glucose and *k* is the insulin clearance rate. The variable β represents beta-cell mass. Following (Topp et al., 2000), β satisfies a differential equation representing the hypothesis that mass adapts homeostatically over a period of years to compensate for insulin resistance.

The variables γ and σ in the *ISR* term represent two aspects of compensation in beta-cell function, respectively, the calcium dependence of exocytosis, as mediated by K(ATP) channels, and the rate of delivery of insulin granules to the plasma membrane. These compensatory variables change on time scales of days to years and are thus effectively constant at their initial conditions during IVGTTs and OGTTs. The details of the equations for β, γ, and σ are not important for this study but are provided in the Supplementary Material—Equations.

**FIGURE 1:**
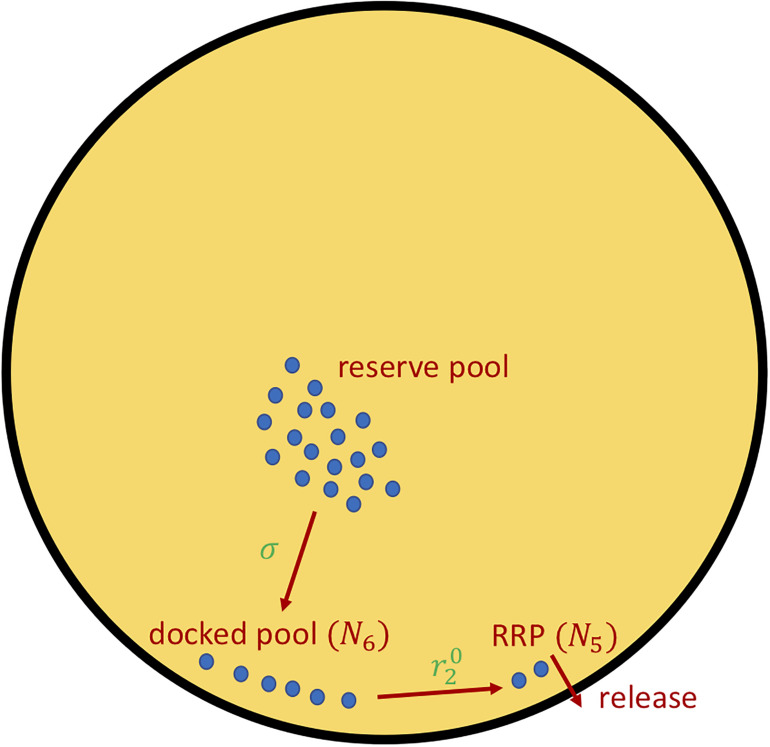
The insulin secretion rate, *ISR* (Eq. 5), can be decomposed into two components, delivery of granules from the reserved pool to the plasma membrane docked pool (variable *N*_6_), with rate proportional to σ (Eqs. 6, 7), and the priming of docked granules into readily releasable pool (RRP; variable *N*_5_) granules, with rate proportional to parameter $r_{2}^{0}$ (Eq. 8). The release steps correspond to the fast calcium-dependent binding steps (variables *N*_1_–*N*_4_) as well as vesicle fusion and insulin release. For details see Eqs. A10–A12, Supplementary Table 8 in Supplementary Material—Equations and refs (Grodsky, 1972; Topp et al., 2000; Dalla Man et al., 2002; Chen et al., 2008; Ha et al., 2016; Ha and Sherman, 2020).

The initial value of σ is varied as a way of increasing AIR. The parameter $r_{2}^{0}$ in the *ISR* controls the rate transfer of insulin vesicles from the docked pool to the readily releasable pool (RRP; Figure 1), known as vesicle priming. This is another way we use to vary AIR. The values of these parameters for each figure are found in Table 1. The details of how σ and $r_{2}^{0}$ determine *ISR* are described next.

**TABLE 1:** Parameters varied to make each figure. The parameter shown in column is the initial value of σ.

Figure, case	S_I_0^–4^ ml/ μU/min	S_G_ (min^–1^)	σ(t = 0) (unitless)	G_F,max_ (unitless)	G_F,b_ (unitless)	${\text{r}}_{2}^{0}$ (s^–1^)
2, control	5.56	0.0118	0.843	0.0	0.285	0.0012
2, large RRP	5.56	0.0104	0.843	0.0	0.285	0.012
3, control 1	1.3888	0.0118	3.374	0.0	0.285	0.0012
3, control 2	2.7776	0.0118	1.687	0.0	0.285	0.0012
3, control 3	4.1664	0.0118	1.127	0.0	0.285	0.0012
3, control 4	5.5552	0.0118	0.843	0.0	0.285	0.0012
3, large RRP 1	1.3888	0.0104	3.374	0.0	0.285	0.012
3, large RRP 2	2.7776	0.0104	1.687	0.0	0.285	0.012
3, large RRP 3	4.1664	0.0104	1.127	0.0	0.285	0.012
3, large RRP 4	5.5552	0.0104	0.843	0.0	0.285	0.012
6, control	5.5552	0.0118	0.843	0.0	0.285	0.0012
6, strong	2.7776	0.0118	1.687	0.0	0.285	0.0012
6, very strong	1.3888	0.0118	3.374	0.0	0.285	0.0012
7, control 1	1.3888	0.0118	3.374	0.0	0.285	0.0012
7, control 2	2.7776	0.0118	1.687	0.0	0.285	0.0012
7, control 3	4.1664	0.0118	1.127	0.0	0.285	0.0012
7, control 4	5.5552	0.0118	0.843	0.0	0.285	0.0012
7, large sigma 1	1.3888	0.0097	6.748	0.0	0.285	0.0012
7, large sigma 2	2.7776	0.0097	3.374	0.0	0.285	0.0012
7, large sigma 3	4.1664	0.0097	2.257	0.0	0.285	0.0012
7, large sigma 4	5.5552	0.0097	1.687	0.0	0.285	0.0012
8 A, B control	5.5552	0.0118	0.843	5.7	0.57	0.006
8 A, B large RRP	5.5552	0.0104	0.843	5.7	0.57	0.06
8 C, D control	5.5552	0.0118	0.843	5.7	0.57	0.006
8 C, D strong	2.7776	0.0118	1.687	5.7	0.57	0.006
8 C, D very strong	1.3888	0.0118	3.374	5.7	0.57	0.006

*For IVGTT, solutions are first equilibrated in basal glucose using OGTT parameters. See section “Materials and Methods” for explanation of each of the parameters.*

**TABLE 2:** List of acronyms, terminology and symbols.

**Physiological Terms**	
T2D	Type 2 Diabetes
MINMOD	Minimal Model
IVGTT	Intravenous Glucose Tolerance Test
OGTT	Oral Glucose Tolerance Test
AIR	Acute Insulin Response to glucose
*S*_*I*_	Insulin sensitivity estimated by MINMOD
DI	Disposition Index, typically *S*_I_*AIR
HIEC	Hyperinsulinemic Euglycemic Clamp
RRP	Readily Releasable Pool (also called primed pool)
Docked pool	Membrane bound vesicles
***Model Terms and Symbols***	
*R_endo_*	Endogenous Glucose Production
*R_exo_*	Exogenously added glucose (orally or intravenously)
*ISR*	Insulin Secretion Rate
Synthetic model	Adaptation of diabetes progression model in Ha and Sherman (2020)
σ	Beta-cell function parameter controlling rate of vesicle docking
$r_{2}^{0}$	Parameter controlling rate of vesicle priming

The insulin secretion rate *ISR* is the output of a model of insulin granule exocytosis (see Figure 1), following broadly the classical two-pool model of Grodsky (1972) as updated and elaborated in Chen et al. (2008) and incorporated in Ha and Sherman (2020) to study the roles of first- and second-phase insulin secretion in diabetes pathogenesis. The key variables in the exocytosis module of the synthetic model are the numbers of vesicles in the docked pool, *N*_6_, and the RRP, *N*_5_ (Figure 1). The rate of transfer of vesicles from the reserve pool (treated as inexhaustible, so not represented by a discrete compartment) is *r*_3_, given by

(6) $$r_{3}=\sigma G_{F}r_{3}^{0}\frac{C_{i}}{C_{i}+K_{p2}}$$

We vary this rate by varying σ, which increases both first- and second-phase secretion because it increases both the docked pool and, by mass action, the RRP. The variable *C_i_* is intracellular calcium and *G_F_* is an increasing function of glucose that represents the effect of one or more mitochondrial metabolites to amplify the efficacy of calcium by increasing vesicle trafficking to the plasma membrane:

(7) GF=GFmax(G-GFsh)kGFαGFkGF+(G-GFsh)kGF+GFb

We view the similar effect of the incretins GLP-1 and GIP to amplify insulin secretion as implicitly folded in to this expression. When we simulate IVGTTs, we reduce the parameters *G*_*F,max*_, and *G*_*F,b*_ in the above equation to account for the greatly reduced effect of incretins during an intravenous glucose challenge.

Finally, the other parameter we use to vary AIR is $r_{2}^{0}$ which controls the rate at which docked vesicles become primed, i.e., transfer from the docked pool to the readily releasable pool (RRP):

(8) $$r_{2}=r_{2}^{0}\frac{C_{i}}{C_{i}+K_{p2}}$$

This increases only first-phase secretion. This rate is also reduced during IVGTTs to reflect reduced incretin effect. For full details of the equations, see Supplementary Material—Equations. For computer code using xpp^1^ and Matlab (MATLAB (2018). *version 9.5.0 (R2018b)*. Natick, Massachusetts: The MathWorks Inc.) that embodies these parameter choices, see Supplementary Material—Matlab Code and xpp Code.

## Results

### Simulated IVGTTs

**FIGURE 2:**
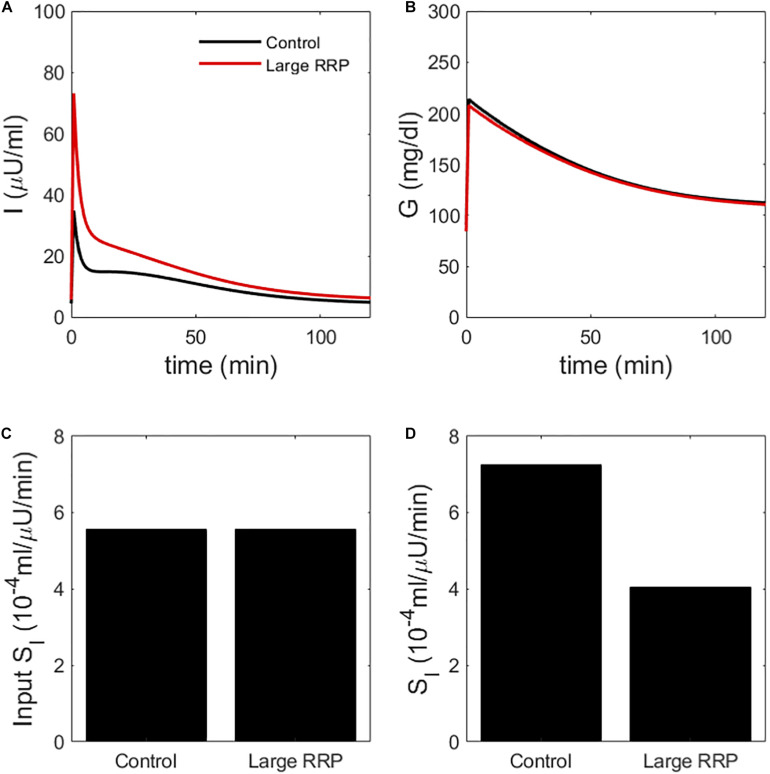
**(A)** insulin and **(B)** glucose during a simulated IVGTT. The red traces represent a case of AIR increased by increasing the rate $r_{2}^{0}$of vesicle priming in the synthetic model (Figure 1). Although the assumed *S*_*I*_ is the same **(C),** MINMOD reports a reduced value **(D).** Control and Large RRP cases differ as well in *S_G_*, which is adjusted to equalize basal glucose. See Table 1 for parameter values for this and subsequent figures.

We create two classes of virtual individuals, control and enhanced AIR, where AIR is increased by increasing the rate of vesicle priming (parameter $r_{2}^{0}$ affecting ISR in Eq. 3), shown in Figure 2. The assumed insulin sensitivity *S*_*I*_ is the same for both cases. The insulin levels and AIR are increased (panel A), whereas the glucose profiles are almost identical (panel B). Figure 2C shows the assumed values of *S*_*I*_, which are the same for both cases. However, MINMOD incorrectly finds lower *S*_*I*_ for the individual with stronger secretion (Figure 2D).

**FIGURE 3:**
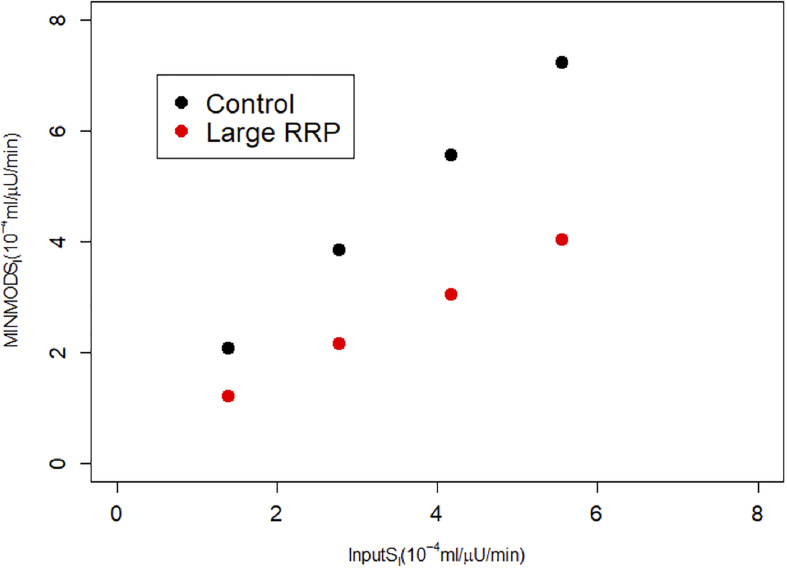
MINMOD estimate of *S*_*I*_ vs. the assumed *S*_*I*_, which is systematically underestimated. Parameters are in Table 1; cases are numbered 1–4 from left to right (increasing *S_I_*) for each of Control and Large RRP.

We repeated the above scenario for several matched pairs of *S*_*I*_ values. Figure 3 shows that MINMOD systematically underestimates *S*_*I*_. Further increasing $r_{2}^{0}$ and AIR results in still lower estimated values of *S*_*I*_ (not shown).

The interpretation by MINMOD of this behavior makes sense: in the high AIR case, insulin is higher, but glucose is not changed much. It therefore concludes that the high AIR individuals are insulin resistant. This is analogous to the Matsuda index of insulin sensitivity, which assumes that insulin sensitivity is inversely proportional to the product of AUC glucose and AUC insulin. Nonetheless, we know the ground truth for these simulations because we prescribed the value of *S*_*I*_, and MINMOD is in disagreement with the assumptions.

### Simulated HIECs

**FIGURE 4:**
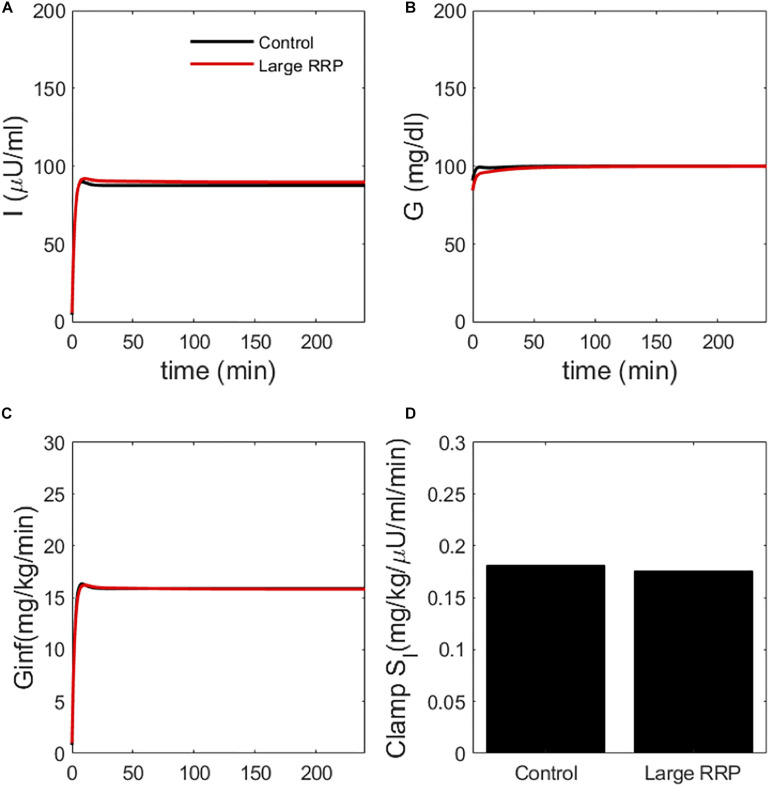
Simulated clamp (HIEC) showing **(A)** insulin, **(B)** glucose, **(C)** glucose infusion rate *G*_*inf*_ and **(D)** insulin sensitivity (Clamp *S*_*I*_) obtained by normalizing infusion rate by insulin. Control and Large RRP have the same parameters as Figure 2.

**FIGURE 5:**
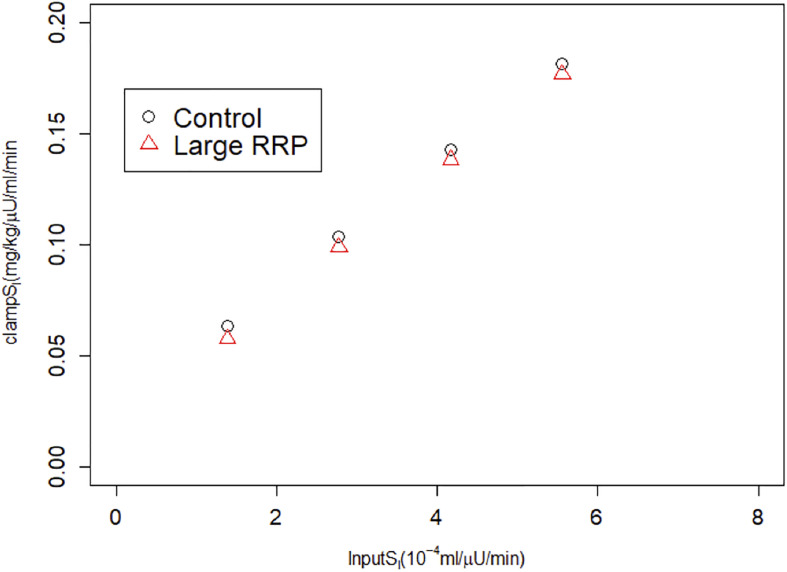
HIEC correctly recovers the assumed *S*_*I*_ independent of RRP size and hence AIR. Cases correspond to Figure 3.

We also simulated HIECs for the same matched pairs of *S*_*I*_ values. One example is illustrated in Figure 4, showing insulin (panel A), glucose (panel B), and the glucose disposal rate normalized for body weight and insulin during the clamp. In contrast to MINMOD, HIEC is indifferent to the RRP size because it does not elicit endogenous insulin secretion and glucose remains near basal levels. Consequently, HIEC correctly estimates *S*_*I*_, independent of AIR (Figure 5).

### Alternative Scenario: Increased Vesicle Docking

**FIGURE 6:**
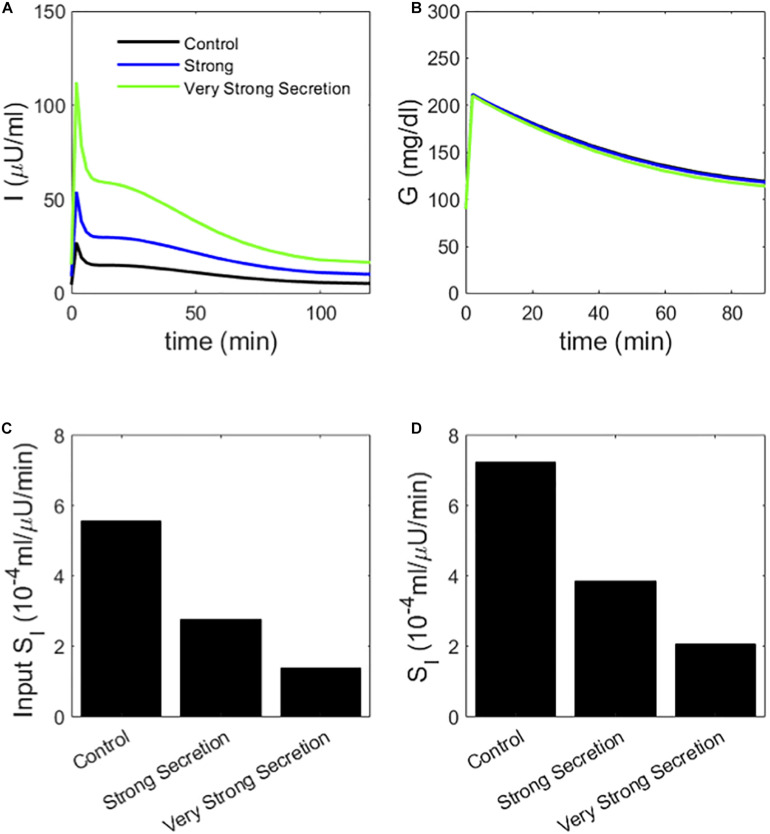
**(A)** insulin and **(B)** glucose during a simulated IVGTT. The blue and green traces represent cases of AIR increased by increasing the rate σ of vesicle docking in the synthetic model. MINMOD reports a reduced *S*_*I*_ **(C)** in agreement with the assumed values **(D).** Cases correspond to Control 1, 2, and 4 in Figure 3.

**FIGURE 7:**
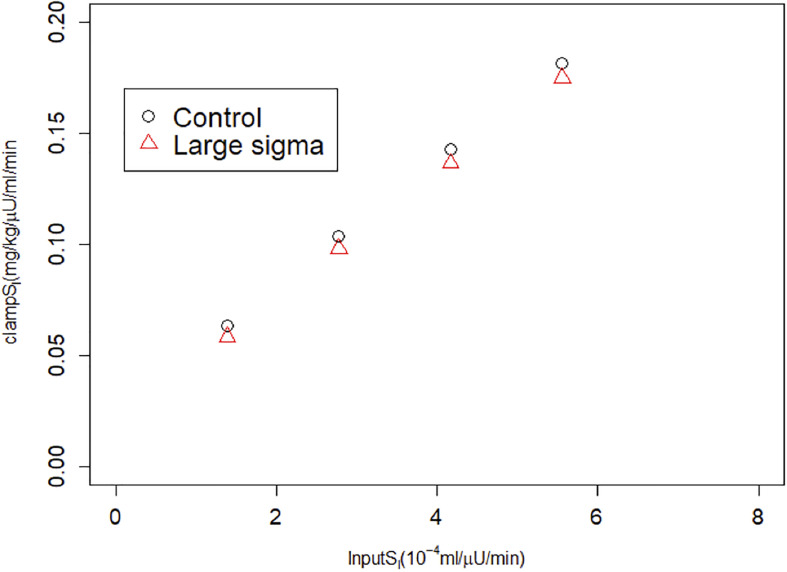
HIEC correctly recovers the assumed *S*_*I*_ independent of vesicle docking rate σ and hence AIR. Control cases correspond to Control 1–4 in Figure 3; Large sigma cases correspond to the same cases but with σ increased 2x.

We next considered an alternative way to attain increased AIR, increasing the rate of vesicle docking (parameter σ in the synthetic model). We proportionally reduced *S*_*I*_ as we increased σ so that this case would correspond to compensatory increases in beta-cell function as insulin sensitivity is reduced. In the simulated IVGTTs (Figure 6), this increased AIR as well as AUC insulin over the entire test (Figure 6A) while keeping the glucose profiles almost unchanged (Figure 6B). In agreement with the assumed values of *S*_*I*_ (Figure 6C), MINMOD in this scenario correctly estimated reductions in *S*_*I*_ inversely proportional to the increased AIR (Figure 6D). This finding is in accord with the definition of insulin resistance—higher insulin with unchanged glucose indicates insulin resistance. HIEC correctly recovers the assumed *S*_*I*_ (Figure 7). Thus, in this scenario MINMOD and HIEC are in agreement.

### Choosing Between the Scenarios

We have illustrated two ways of increasing AIR, increased vesicle priming and increased vesicle docking. In the former, MINMOD underestimates *S*_*I*_, whereas in the latter, MINMOD’s estimates are correct.

We are left with the question of which scenario is more relevant for the case of comparing Black and White individuals, for which we do not know the ground truth regarding insulin sensitivity. We address this by looking at the performance of Black and White individuals on another test, the OGTT. Clinical studies show that Blacks, when normally glucose tolerant, have slightly lower glucose levels and somewhat higher insulin levels than Whites (Weiss et al., 2006; Chung et al., 2019; Fosam et al., 2020).

**FIGURE 8:**
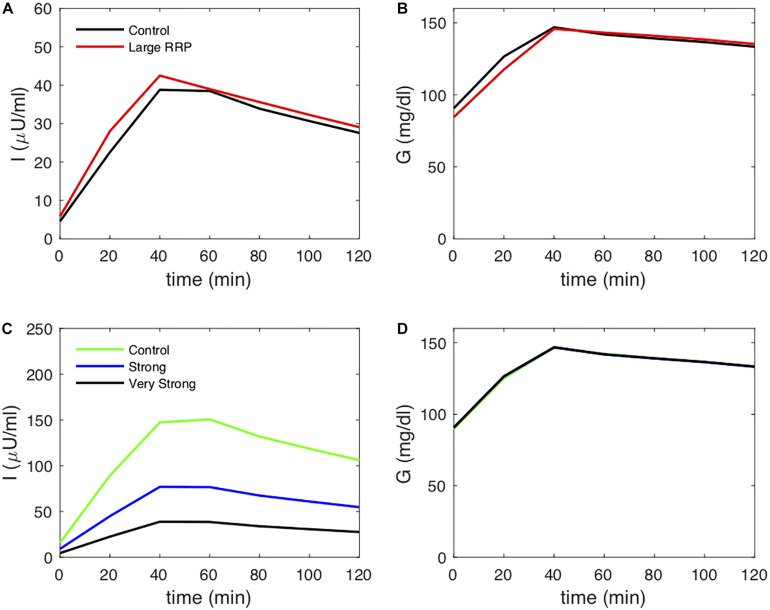
Insulin **(A)** and glucose **(B)** during OGTTs simulated with the synthetic model, using the parameters of Figure 2 (increased RRP case) but with incretin effect included (larger values of *G*_*F,max*_, and *G*_*F,b*_ in Eq. 7 and $r_{2}^{0}$ in Eq. 8). Insulin **(C)** and glucose **(D)** during OGTTs simulated with the synthetic model, using the parameters of Figure 6 but with incretin effect included.

We used the synthetic model to simulate OGTTs for the scenario of increased vesicle priming (Figures 8A,B). The insulin (panel A) and glucose (panel B) profiles are similar, with the high AIR individuals exhibiting slightly higher insulin and slightly lower glucose. This is in agreement with some but not all clinical observations in Blacks and Whites; see Discussion. The natural interpretation of the OGTT is that insulin sensitivity of the two hypothetical individuals is similar. This is in contrast with the simulated IVGTTs, in which the high AIR individual had higher insulin but similar glucose levels (Figure 2).

We also simulated OGTTs for the scenario of increased vesicle docking (Figures 8C,D). The insulin (panel C) is higher for the AIR individuals, whereas the glucose profiles are the same (panel D), the same pattern seen in the IVGTT (Figure 6). The natural interpretation of both the OGTT and the IVGTT for this scenario is that the high AIR individual is more insulin resistant. However, the behavior during the OGTT is in agreement with some observations in Black and White individuals, especially at the later time points. Specifically, the hypothetical high AIR individuals simulated here have much higher glucose at the 2-h timepoint, as found in some studies (Osei and Schuster, 1994; Osei et al., 1997).

## Discussion

Our motivation for this study was an interest in resolving discrepancies between IVGTT and HIEC in estimating insulin sensitivity of Black and White individuals. IVGTT, interpreted using MINMOD, generally finds that Blacks have lower insulin sensitivity, whereas HIEC generally does not find differences (Saad et al., 1991; Stefan et al., 2004; Pisprasert et al., 2013; Ebenibo et al., 2014; Bello et al., 2019). We asked whether MINMOD is misled by the higher AIR during IVGTTs of the Black subgroup to underestimate insulin sensitivity, *S*_*I*_. We investigated this question with a synthetic model (Ha et al., 2016; Ha and Sherman, 2020) to generate hypothetical individuals with varying degrees of AIR and *S*_*I*_ and simulate their performance during IVGTTs and HIECs.

We considered two scenarios for increased AIR. In one, high AIR was the result of increased size of the RRP, which is closely related to first-phase insulin secretion, and one in which high AIR was the result of increased rate of mobilization of insulin granules, which increases both first- and second-phase secretion. In both scenarios, the simulated IVGTTs were qualitatively similar to those exhibited by high AIR and low AIR individuals. However, the first way resulted in a systematic underestimation of *S*_*I*_ by MINMOD, that is, lower than the assumed value. The second way resulted in a correct recovery of *S*_*I*_ by MINMOD. HIEC by contrast recovered *S*_*I*_ equally well in both cases, independent of AIR. The question then is which scenario corresponds better to the experimentally observed differences between Black and White groups. Published OGTT data vary, likely depending on the age, BMI, sex (including menopausal status) and other characteristics of the population studied, as well as how well the groups are matched. Weiss et al. (2006) showed similar insulin and glucose profiles in Blacks and Whites, consistent with the RRP scenario, whereas Osei and colleagues (Osei and Schuster, 1994; Osei et al., 1997) showed similar glucose but substantially elevated insulin during OGTT in Blacks, consistent with the second scenario. We conclude that a finding using MINMOD of lower *S*_*I*_ in Blacks relative to Whites, or any comparison of high and low AIR groups, should be interpreted cautiously in the absence of corroborating evidence from OGTTs or clamps.

It is instructive to view the issue treated here in terms of MINMOD’s response to changes in AUC insulin as well as AIR. The Matsuda index for OGTTs defines insulin sensitivity as inversely related to the product of AUC insulin and AUC glucose. MINMOD estimation of *S*_*I*_ in the scenarios considered here is likewise inversely related to the product of AUC insulin and AUC glucose. In one scenario, that inverse relationship correctly corresponds to the assumed physiology, in the other it is incorrect. We hasten to point out, however, that MINMOD estimates of *S*_*I*_ are not necessarily inversely related to AUC insulin. An example of great importance for the way MINMOD is generally implemented is the effect of infusing exogenous insulin at the 20-minute time point of the IVGTT. This modification, the insulin-modified IVGTT or IM-IVGTT, was introduced to improve estimates for individuals with greatly reduced endogenous secretion, such as those with type 1 diabetes or advanced type 2 diabetes. Estimates of *S*_*I*_ obtained with the standard IVGTT and the IM-IVGTT are comparable, but the IM-IVGTT has greater precision, as shown in, for example (Quon et al., 1994; Pacini et al., 1998). Yang et al. (1987) showed similarly that increasing insulin secretion at the 20-minute time point by injecting tolbutamide or delaying the peak of insulin by injecting somatostatin does not change the estimated value of *S*_*I*_ but reduces the error of the estimate. Thus, the response of MINMOD to changes in AUC insulin depends strongly on the context.

The context that we are concerned with here is whether MINMOD or HIEC is more trustworthy in evaluating ethnic differences. In the scenarios we considered, for which *S*_*I*_ was known *a priori*, we found that HIEC was more trustworthy. It is also important to emphasize that HIEC is a method that directly measures insulin sensitivity whereas the minimal model uses a simulation approach. The application to studies of Black and White cohorts depends then on whether either of our scenarios describes correctly the underlying mechanism for enhanced AIR in black individuals. A PKPD studies using IVGTT suggests that the scenario of increased first-phase secretion due to larger RRP is a better representation (Xie et al., 2010). Of course, other putative mechanisms that we have not considered may be even better. We conclude that, at minimum, caution should be exercised in interpreting MINMOD estimates of *S*_*I*_ between populations that differ substantially in AIR such as Blacks and Whites.

## Data Availability Statement

The original contributions presented in the study are included in the article/Supplementary Material, further inquiries can be directed to the corresponding author/s.

## Author Contributions

JH carried out the simulations. JH, RM, AS, and MQ contributed to the design of the study, the analysis and interpretation of the results, and the writing. All authors approved the final version of the manuscript.

## Conflict of Interest

The authors declare that the research was conducted in the absence of any commercial or financial relationships that could be construed as a potential conflict of interest.

## Footnotes

1 http://www.math.pitt.edu/b̃ard/xpp/xpp.html
