# Supplementary material for: When MINMOD Artifactually Interprets Strong Insulin Secretion as Weak Insulin Action
Source: Front Physiol. 2021 Apr 22;12:601894. doi: 10.3389/fphys.2021.601894 (PMC8100339; doi:10.3389/fphys.2021.601894)
Supplement: Supplementary file 1 [file Data_Sheet_1.zip › Supplementary Material - Equations V3.pdf]

Supplemental Material (Equations and Parameter Tables) for "MINMOD Artifactually Interprets Strong Insulin Secretion as Weak Insulin Action," by J. Ha, R. Muniyappa, A. S. Sherman, and M. J. Quon

The model is the same as in Ha and Sherman, Type 2 Diabetes: One Disease, Many Pathways, Am. J. Physiol. (Endocrinol. and Metab.), **319**(2):E410-E426 (doi: https://journals.physiology.org/doi/abs/10.1152/ajpendo.00512.2019) except as noted below in red. The changes were (1) addition of an interstitial insulin compartment (X) for to better represent IVGTT dynamics and minor adjustment of some parameters for glucose amplification (Table S7) and vesicle mobilization (Table S8).

$$\frac{dG}{dt} = GFLUX + HGP - (S_G + X)G$$

$$\frac{dX}{dt} = -[X - S_I(I - I_b)]/p_2$$

$$\frac{dI}{dt} = \frac{\beta}{V} ISR - kI$$

$$\frac{d\beta}{dt} = \frac{(P(ISR) - A(M))\beta}{\tau_\beta}$$

$$\frac{d\gamma}{dt} = \frac{\gamma_\infty(G) - \gamma}{\tau_\gamma}$$

$$\frac{d\sigma}{dt} = \frac{\sigma_\infty(ISR, M) - \sigma}{\tau_\sigma}$$

Where $GFLUX = OGTT\ or\ IVGTT$ below. Also: S_G= 0.0118 min^{-1} (was previously denoted E_{G0}), V = 7200 mL, p_2 = 720 min, and k = 0.486 min^{-1} (other parameters defined in tables below).

MEAL MODEL (not used in this study but retained to preserve equation and table numbering)

$$MEAL = meal_{max}\left(\frac{t^{mk}}{\alpha_{meal}+t^{mk}}\right)^{\eta} \exp(-\mu t) \qquad \text{(A1)}$$

(the same as Eq. 8 in the main text of [1]). Figs. A3 A, B show glucose fluxes and the corresponding glucose concentration in response to three meals per day.

Table S1. Parameters for *MEAL*

Parameter Name	Value	Description	Unit
$meal_{max}$	11.055	Maximum meal rate	*mg/dl/min*
mk	4	Degree of polynomial	unitless
α_{meal}	40	Half activation value	unitless
η	0.3	Exponent of rise for meal flux	unitless
μ	0.015	Rate of decay for meal flux	unitless

OGTT MODEL

Figs. A3 C, D show glucose fluxes and the corresponding glucose response during an OGTT. The OGTT fluxes are modeled by a piecewise linear function similar to [2]. Specifically, the term *GFLUX* in the *G* equation is replaced by *OGTT*, where

$OGTT = \frac{OGTT_0}{V_G}$, where $V_G = BW \times \bar{V}$ is the volume of distribution for glucose in dL, BW is body weight in kg, $\bar{V}$= 1.569 dL/kg [3], and

$$OGTT_0 = a_{i-1} + \frac{a_i - a_{i-1}}{t_i - t_{i-1}}(t - t_{i-1}), t_{i-1} < t < t_i, i = 1,2,3 \quad \text{(A2)}$$

$OGTT_o = 0$, elsewhere

The simulations in the paper were carried out assuming *BW* = 75 kg, for a volume of distribution of 11.77 L, but this formulation makes the glucose load 75 kg independent of those choices. The values for a_i are given in Table S2.

Table S2. Parameters for *OGTT*

Parameter name	Value	Description	Unit
a_0	0	Glucose flux at $t=t_0$	*mg/min*
a_1	588.4	Glucose flux at $t=t_1$	*mg/min*
a_2	353	Glucose flux at $t=t_2$	*mg/min*

a_3	0	Glucose flux at $t=t_3$	*mg/min*
t_0	0	Initial time of activation of glucose flux	*min*
t_1	15	First time of activation of glucose flux	*min*
t_2	120	Second time of activation of glucose flux	*min*
t_3	240	Third time of activation of glucose flux	*min*

IVGTT MODEL.

The rapid rise and decline in glucose flux are modeled by a polynomial and exponential function as shown in Figs. 6A, B, and C. The term *GFLUX* in the *G* equation is replaced by *IVGTT*, where

$$IVGTT = \frac{IVGTT_{bar}}{BW\,V_G} t^{\kappa} \exp(-\lambda t) \qquad \text{(A3)}$$

and $IVGTT_{bar}$ is given in Table S3 along with the other parameters. As for the OGTT, the simulations were carried out assuming BW = 75kg.

Table S3 Parameters for *IVGTT*

Parameter name	Value	Description	Unit
$IVGTT_{bar}$	1.1032×10^8	Maximum glucose flux	*mg/min*
κ	1	Degree of polynomial	unitless
λ	10	Decay rate of exponential function	1/min

Hepatic Glucose Production (HGP). HGP is modeled as a function of insulin concentration I and peripheral insulin sensitivity S_I using equations A4, A5, and A6. The model HGP is a decreasing function of I (Eq. A4): As I increases during the post-prandial state, HGP is suppressed (Fig. A4A). $hepa_{max}$ and α_{HGP} are modeled as decreasing functions of peripheral insulin sensitivity S_I, as shown in Figs. A4 B, C (the same as equations 9 – 11 in the main text of [1]).

$$HGP = \frac{hepa_{max}}{\alpha_{HGP} + hepa_{SI}I} + HGP_{bas} \quad \text{(A4)}$$

$$hepa_{max} = \frac{hepa_{bar}}{hepa_k + S_I} + hepa_{sh} \quad \text{(A5)}$$

$$\alpha_{HGP} = \frac{\alpha_{bar}}{\alpha_k + S_I} + \alpha_{sh} \quad \text{(A6)}$$

Table S4 Parameters for *HGP*

Parameter name	Value	Description	Units
HGP_{bas}	0.104	Basal HGP rate	*mg/dl/min*
$hepa_{bar}$	15.443	Coefficient of $hepa_{max}$	*10^{-4}min(mg/dl/min)*
$hepa_k$	0.27	Translation of $hepa_{max}$	*10^{-4}ml/μU/min*
$hepa_{sh}$	–3.542	Shift of $hepa_{max}$	*(μU/ml)(mg/dl/min)*
α_{bar}	6	Coefficient of α_{HGP}	*10^{-4}/min*
α_k	0.4	Translation of α_{HGP}	*10^{-4}ml/μU/min*
α_{sh}	-0.5	Shift of α_{HGP}	*μU/ml*

Bulk Cytosolic Calcium Concentration (C_i) Model.

$$C_i = \frac{C_{i_{max}}(M+\gamma)^{kC_i}}{\alpha_{C_i}{}^{kC_i} + (M+\gamma)^{kC_i}} + C_{i_b} \quad \text{(A7)}$$

Table S5 parameters for Ca^{2+} model

Parameter name	Value	Description	Unit
$C_{i,max}$	2	Maximum Ca^{2+}	*μM*
kC_i	4	Exponent of sigmoidal function	unitless
α_{Ci}	0.62	Half activation value	unitless

$C_{i,b}$	0.07	Basal calcium	μM

Microdomain Ca^{2+} Concentration (C_{md}) Model

$$C_{md} = \frac{C_{md_{max}}(C_i)^{kC_{md}}}{\alpha_{C_{md}}{}^{kC_{md}} + (C_i)^{kC_{md}}} + C_{md_b} \qquad \text{(A8)}$$

Table S6 Parameters for Microdomain

Parameter name	Value	Description	Unit
$C_{md,max}$	150	Maximum C_{md}	μM
kC_{md}	4	Exponent of sigmoidal function	unitless
α_{Cmd}	1	Half activation value	unitless
$C_{md,b}$	0.0635	Basal microdomain calcium	μM

Glucose Amplifying Factor Model.

$$G_F = \frac{G_{F_{max}}(G - G_{F_{sh}})^{kG_F}}{\alpha_{G_F}{}^{kG_F} + (G - G_{F_{sh}})^{kG_F}} + G_{F_b} \qquad \text{(A9)}$$

G_F implicitly includes a component corresponding to the incretin effect. For IVGTT and hyperglycemic clamp simulations, we reduced $G_{F_{max}}$ by a factor of 10, which reduces G_F by a factor of about 2.

Table S7 Parameters for amplifying factor.

Parameter name	Value OGTT (IVGTT)	Description	Unit
$G_{F,max}$	5.71 (0)	Maximum value	unitless
$G_{F,sh}$	–89	Shift of Glucose	*mg/dl*
kG_F	30	Exponent of sigmoidal function	unitless
$\alpha_{G,F}$	260	Half activation	*mg/dl*
$G_{F,b}$	0.57(0.285)	Basal G_F	unitless

Insulin Granule Exocytosis Model. The model was previously published in [4]. The rate of mobilization of insulin granules, r_3, is modified to include a component of β-cell function, σ, and a glucose amplifying factor G_F that is modeled by a Hill function, whereas it was originally modeled by a step function in [4].

$$N_{1C} = \frac{km_1}{3k_1 C_{md} + rm_1}$$

$$N_{1D} = \frac{r_1}{3k_1 C_{md} + rm_1}$$

$$N_{2E} = \frac{3k_1 C_{md}}{2k_1 \ C_{md} + km_1}$$

$$N_{2F} = \frac{2km_1}{2k_1 \ C_{md} + km_1}$$

$$N_{3L} = \frac{2k_1 C_{md}}{2km_1 \ + \ k_1 C_{md}}$$

$$N_{3N} = \frac{3km_1}{2km_1 \ + k_1 C_{md}} \qquad \text{(A10)}$$

$$C_{N4} = \frac{k_1 C_{md}}{2km_1 \ + \ u_1}$$

$$C_{N3} = \frac{N_{3L}}{1 - N_{3N} C_{N4}}$$

$$C_{N2} = \frac{N_{2E}}{1 - N_{2F C_{N3}}}$$

$$C_{N1} = \frac{N_{1D}}{1 - N_{1C}C_{N2}}$$

$$\frac{dN_5}{dt} = t_s(rm_1 C_{N1} N_5 - (r_1 + rm_2)N_5 + r_2 N_6)$$

$$\frac{dN_6}{dt} = t_s(r_3 + rm_2 N_5 - (rm_3 + r_2)N_6)$$

where

$$r_3 = \sigma G_F r_3^0 \frac{C_i}{C_i + k_{p2}} \qquad \text{(A11)}$$

and σ is determined by Eqs. (6) in the main text of [1] and Eq. (A16) below.

Equations of the exocytosis model, simplified from [4] by setting fast steps $N_1 - N_4$ to steady state:

$$N_1 = C_{N1} N_5$$

$$N_2 = C_{N2} N_1$$

$$N_3 = C_{N3} N_2$$

$$N_4 = C_{N4} N_3$$

$$N_F = u_1 N_4 / u_2$$

$$N_R = N_F u_2 / u_3$$

$$r_2 = r_2^0 \frac{C_i}{C_I + k_{p2}} \qquad \text{(A12)}$$

$$ISR = \rho t_s u_3 N_R$$

The output of the exocytosis model vesicles/min/cell. In contrast to the first version of the model [5], σ is a unitless scale factor modifying vesicle delivery to the plasma membrane, so we need the factor ρ to convert vesicles/cell/min to μU(insulin)/mg(beta cells)/min for substitution of *ISR* into Eq. 2 for *I*. The value of ρ is approximately 90 μU/mg based on the following estimates:

1 vesicle has 9 fg of insulin [6].

A beta cell has a volume of about 3 pL [7] and weighs about 2.9 ng, so there are about 3.5×10^5 cells/mg.

1 U = 0.035 mg of insulin, giving 3.5×10^4 fg/μU.

Combining these three factors gives ρ = 90 μU/mg. However, we find that this rate, based on single-cell measurements of capacitance, gives whole-body insulin secretion that is too large. Previous reports have said that islet secretion in vivo is 3-fold [7] and in vitro 25-fold [8] smaller than single-cell secretion. The inhibition of insulin secretion by paracrine somatostatin and by insulin itself may contribute to this. We have compromised and reduced ρ 10-fold to 9 μU/mg. With this choice, total secretion rate ($\beta\, ISR/V$) is in the range 100 – 550 pmol/min during an OGTT, compared to 200 – 700 pmol/min in experimental data from 7 NGT and 4 IGT subjects [9], and peak post-prandial secretion for an NGT subject is about 0.6 ng/ml/min, close to results from a mixed meal test in [10].

Table S8 Parameters for the exocytosis model

Parameter name	Value (IVGTT)	Description	Unit
t_s	60	Unit conversion factor	*sec/min*
k_1	20	Flux	$(\mu M\ sec)^{-1}$
km_1	100	Flux	sec^{-1}
r_1	0.6	Flux	sec^{-1}
r_2^0	0.006 (0.0012)	Flux	sec^{-1}
rm_2	0.001	Flux	sec^{-1}
r_3^0	2.0	Flux	sec^{-1}
u_1	2000	Flux	sec^{-1}
u_2	3	Flux	sec^{-1}
u_3	0.02	Flux	sec^{-1}
k_{p2}	2.3	Half activation value	μM

Dynamics of peripheral and hepatic insulin resistance. Both peripheral insulin sensitivity, S_I, and hepatic insulin resistance, $hepa_{IR}$, are modeled as decreasing exponentially to target levels tar_{SI} and tar_{hepaIR} with time constants τ_{SI} and τ_{hepaIR}, respectively.

$$\frac{dSI}{dt} = \frac{t_{min}(tar_{SI} - SI)}{\tau_{SI}} \quad \text{(A13)}$$

$$\frac{dhepa_{SI}}{dt} = \frac{t_{min}(tar_{hepa_{SI}} - hepa_{SI})}{\tau_{hepa_{SI}}} \quad \text{(A14)}$$

Parameter adjustments for daily glucose responses. Some parameters in Tables S9 and S10 have been changed from [5] to accommodate daily glucose fluctuations.

γ–dynamics:

$$\gamma_{\infty}(G) = \frac{\gamma_{max}}{1+exp((G-\gamma_s)/\gamma_n)_0} \quad \text{(A15)}$$

Table S9. Parameter adjustments of γ–dynamics for daily glucose fluctuations

Parameter name	Value	Description	Unit
γ_{max}	0.4	Maximum value of γ	unitless
γ_s	100	Default value of horizontal shift	*mg/dl*
γ_n	5	Slope factor of γ_{∞}	unitless
γ_0	0.2	Baseline value of γ	unitless
τ_{γ}	3,081.6	Time constant of γ	min (2.14 *d*)

σ–dynamics:

$$\sigma_{\infty}(ISR, M) = \sigma_{ISR\infty}(ISR)\sigma_{M\infty}(M) + \sigma_b$$

$$\sigma_{ISR\infty}(ISR) = \frac{\sigma_{ISRmax}}{1+\sigma_{ISRk}\exp\left(-\frac{ISR-\sigma_{ISRs}}{\sigma_{ISRn}}\right)} \quad \text{(A16)}$$

$$\sigma_{M\infty}(M) = 1 - \frac{\sigma_{Mmax}}{1 + \sigma_{Mk}\exp\left(-\frac{M_\sigma - \sigma_{Ms}}{\sigma_{Mn}}\right)}$$

where $M_\sigma = M(G - G_{\sigma s})$

Table S10. Parameters of σ-dynamics for daily glucose fluctuations

Parameter name	Value	Description	Unit
σ_{ISRmax}	1.4	Maximum value of $\sigma_{ISR\infty}$	$\frac{\mu U}{\mu g \cdot d}$
σ_{ISRk}	1	Weight of $\sigma_{ISR\infty}$	
σ_{ISRs}	0.1	Horizontal shift of $\sigma_{ISR\infty}$	
σ_{ISRn}	0.1	Slope of $\sigma_{ISR\infty}$	
σ_b	0.0175	Basal sigma value	$\frac{\mu U}{\mu g \cdot d}$
σ_{Mmax}	1	Scale factor of $\sigma_{M\infty}$	
σ_{Mk}	0.2	Weight of $\sigma_{M\infty}$	
σ_{Ms}	0.2	Horizontal shift of $\sigma_{M\infty}$	$\frac{\mu U}{\mu g \cdot d}$
σ_{Mn}	0.02	Slope of $\sigma_{M\infty}$	
$G_{\sigma s}$	35	shift of glucose dependent M	*mg/dl*
τ_σ	3.598×10^5	Time constant of σ	*min* (249.9 *d*)

β–dynamics:

$$P(ISR) = P_{max}\frac{ISR^{kP}}{{\alpha_P}^{kP} + ISR^{kP}} \quad \text{(A17)}$$

$$A(M) = A_{max} \frac{M^{kA}}{{\alpha_A}^{kA} + M^{kA}}$$

Table S11. Parameters of β-dynamics for daily glucose fluctuations

Parameters	Values	Description	Units (unitless if blank)
k_M	2	Exponent of metabolic rate *M*	
α_M	150	Half maximum *G* value for *M*	*mg/dl*
$kISR$	2	Exponent of insulin secretion rate *ISR*	
α_{ISR}	1.2	Half maximum *M* value for *ISR*	
P_{max}	4.55	Maximum proliferation rate	*1/day*
kP	4	Exponent of proliferation rate	
α_P	41.77	Half maximum value of *ISR* for proliferation	
A_{max}	3.11	Maximum apoptosis rate	*1/day*
kA	6	Exponent of apoptosis rate	
α_A	0.44	Half maximum value of *M* for apoptosis	
A_b	0.8	Basal apoptosis rate	*1/day*
τ_β	1.008×10^7	Time constant of β	min (7000 *d*)

[1] J. Ha, and A. Sherman, Type 2 diabetes: one disease, many pathways. Am J Physiol Endocrinol Metab 319 (2020) E410-E426.
[2] C. Dalla Man, A. Caumo, and C. Cobelli, The oral glucose minimal model: estimation of insulin sensitivity from a meal test. IEEE Trans Biomed Eng 49 (2002) 419-29.
[3] A. Mari, G. Pacini, E. Murphy, B. Ludvik, and J.J. Nolan, A model-based method for assessing insulin sensitivity from the oral glucose tolerance test. Diabetes Care 24 (2001) 539-48.
[4] Y.D. Chen, S. Wang, and A. Sherman, Identifying the targets of the amplifying pathway for insulin secretion in pancreatic beta-cells by kinetic modeling of granule exocytosis. Biophys J 95 (2008) 2226-41.
[5] J. Ha, L.S. Satin, and A.S. Sherman, A Mathematical Model of the Pathogenesis, Prevention, and Reversal of Type 2 Diabetes. Endocrinology 157 (2016) 624-35.
[6] P. Rorsman, and E. Renstrom, Insulin granule dynamics in pancreatic beta cells. Diabetologia 46 (2003) 1029-45.
[7] P. Rorsman, and M. Braun, Regulation of insulin secretion in human pancreatic islets. Annu Rev Physiol 75 (2013) 155-79.
[8] S. Gopel, Q. Zhang, L. Eliasson, X.S. Ma, J. Galvanovskis, T. Kanno, A. Salehi, and P. Rorsman, Capacitance measurements of exocytosis in mouse pancreatic alpha-, beta- and delta-cells within intact islets of Langerhans. J Physiol 556 (2004) 711-26.
[9] E. Breda, M.K. Cavaghan, G. Toffolo, K.S. Polonsky, and C. Cobelli, Oral glucose tolerance test minimal model indexes of beta-cell function and insulin sensitivity. Diabetes 50 (2001) 150-8.
[10] S.T. Chung, M. Galvan-De La Cruz, P.C. Aldana, L.S. Mabundo, C.W. DuBose, A.U. Onuzuruike, M. Walter, A.M. Gharib, A.B. Courville, A.S. Sherman, and A.E. Sumner, Postprandial Insulin Response and Clearance Among Black and White Women: The Federal Women's Study. The Journal of clinical endocrinology and metabolism 104 (2019) 181-192.
